# Unpredicted Sudden Death due to Recurrent Infratentorial Hemangiopericytoma Presenting as Massive Intratumoral Hemorrhage: A Case Report and Review of the Literature

**DOI:** 10.1155/2014/230681

**Published:** 2014-11-16

**Authors:** Toshihide Tanaka, Naoki Kato, Yuzuru Hasegawa, Yuichi Murayama

**Affiliations:** ^1^Department of Neurosurgery, Jikei University School of Medicine Kashiwa Hospital, 163-1 Kashiwashita, Kashiwa, Chiba 277-8567, Japan; ^2^Department of Neurosurgery, Jikei University School of Medicine, 3-25-8 Nishishimbashi, Minato-ku, Tokyo 105-8461, Japan

## Abstract

Unpredicted sudden death arising from hemangiopericytoma with massive intracranial hemorrhage is quite rare. We encountered a patient with recurrent infratentorial hemangiopericytoma presenting as life-threatening massive intracerebral hemorrhage. A 43-year-old man who had undergone craniotomy for total resection of an infratentorial hemangiopericytoma 17 months earlier presented with morning headache and generalized convulsions. Computed tomography revealed a massive hematoma in the right infratentorial region causing tonsillar herniation and emergency surgery was performed to evacuate the hematoma. Histological findings revealed hemangiopericytoma with hemorrhage. Neurological status remained unimproved and brain death was confirmed postoperatively. Hemangiopericytoma presenting as massive hemorrhage is quite rare. Since the risk of life-threatening massive hemorrhage should be considered, careful postoperative long-term follow-up is very important to identify tumor recurrences, particularly in the posterior cranial fossa, even if the tumor is completely removed.

## 1. Introduction

Hemangiopericytoma is a highly vascular tumor arising from pericytes. This tumor occasionally shows malignant behaviors such as local recurrence or distant metastasis [1]. Brain tumor manifesting as intracranial hemorrhage is a well-known phenomenon [2], but hemangiopericytoma is rarely associated with hemorrhage in either central nervous system or extracranial sites [3–5]. We encountered an extremely rare case with recurrent infratentorial hemangiopericytoma presenting as life-threatening massive intracranial hemorrhage.

## 2. Case Report

**Figure 1:**
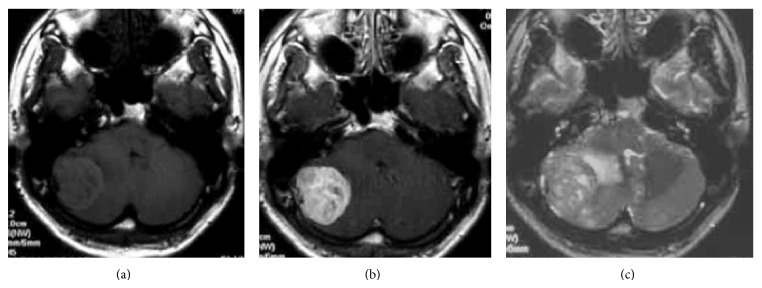
Preoperative T1-weighted plain (a), gadolinium-enhanced (b), and T2-weighted (c) magnetic resonance imaging showing the infratentorial extra-axial tumor with perifocal edema. Note that the tumor shows strong, homogeneous enhancement.

**Figure 2:**
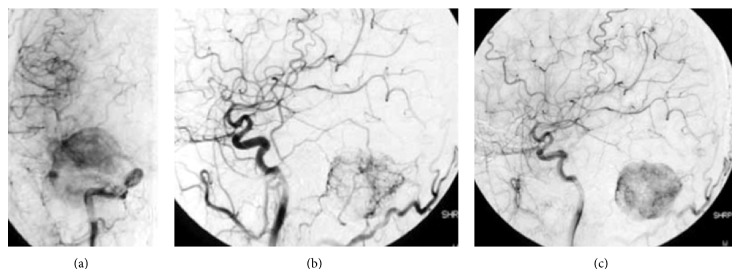
Right carotid angiography (CAG) revealing microvascular networks in the tumor. Anterior-posterior (a) and lateral (b) CAG demonstrates the tumor being fed by the ascending pharyngeal artery and occipital artery.

**Figure 3:**
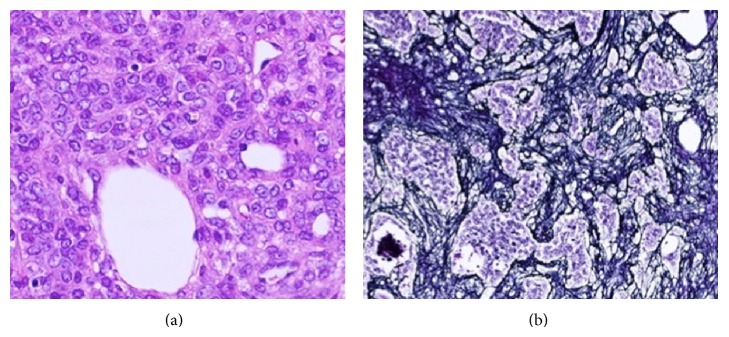
Photomicrographs revealing a highly cellular tumor consisting of oval and spindle-shaped cells surrounding slit-like, elongated blood vessels with a “staghorn” appearance ((a): ×200). A rich network of reticulin fibers is present among the tumor cells ((b): ×200).

**Figure 4:**
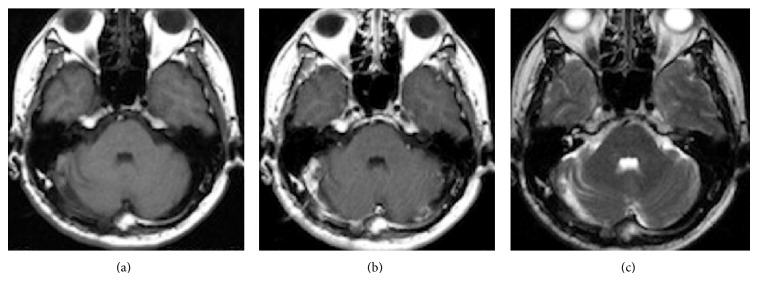
Postoperative T1-weighted plain (a), gadolinium-enhanced (b), and T2-weighted (c) MRI with contrast medium suggesting complete removal of the tumor without recurrence at 9 months postoperatively.

**Figure 5:**
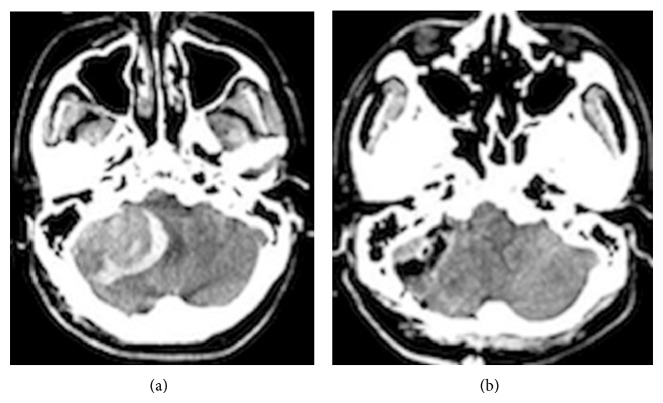
Computed tomography (CT) revealing infratentorial tumor with massive hematoma around the tumor, compressing the brainstem (a). Postoperative CT shows complete removal of the tumor and hematoma (b).

**Figure 6:**
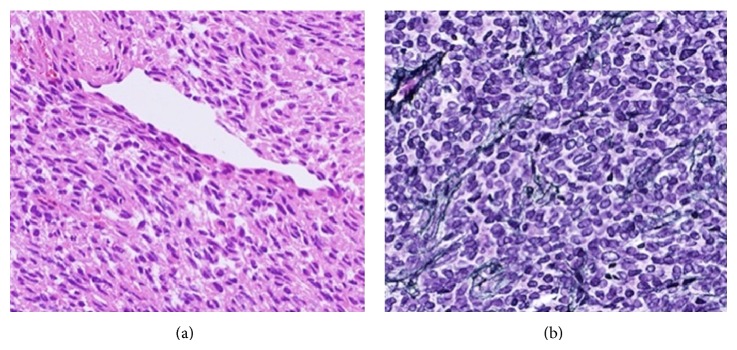
Photomicrographs revealing a highly cellular tumor consisting of spindle cells surrounding elongated blood vessels with a “staghorn” appearance ((a): ×200). A rich network of reticulin fibers is present among the tumor cells ((b): ×200).

A 42-year-old man presented with morning headache. Magnetic resonance imaging (MRI) showed the mass as hypointense on T1-weighted imaging and hyperintense on T2-weighted imaging, with homogenous enhancement on gadolinium-enhanced imaging (Figure 1). Cerebral angiography demonstrated that the tumor was fed by the ascending pharyngeal and occipital arteries (Figures 2(a)–2(c)). Surgery was performed via a retrosigmoid lateral suboccipital approach. Gross total removal of the tumor was performed and the dural attachment was cauterized. The tumor was highly vascular, with elastic, hard, and soft reddish components. Histological examination showed that the tumor was densely cellular and was composed of oval-shaped cells with vascular spaces (Figure 3(a)). A rich network of reticulin fibers was seen between the tumor cells and vascular spaces (Figure 3(b)). MIB-1 index was 3%. The postoperative course was uneventful. Postoperative MRI showed total removal of the tumor, and the patient was discharged 1 week postoperatively. Eight months later, follow-up MRI demonstrated that no recurrence was apparent except enhancing lesion on the convexity dura at the previous excision site that is retrospectively considered to be suspicious of a recurrence (Figure 4). Although the neurological status of the patient had been intact at that time, nine months after the final follow-up MRI, the patient presented with sudden onset of headache and generalized convulsions, followed by cardiorespiratory arrest. Computed tomography (CT) revealed a huge hematoma in the resected tumor cavity that resulted in cerebellar tonsillar herniation (Figure 5); therefore, emergent craniotomy was performed. The hematoma was located in the subdural space and contained soft, reddish extra-axial tumor tissue with abundant vasculature. Histological examination showed a highly cellular tumor composed of spindle cells surrounding elongated blood vessels with a “staghorn” appearance (Figure 6). MIB-1 index was 5%. Postoperatively, the clinical course was dismal, and two weeks postoperatively, a clinical diagnosis of brain death was confirmed. Autopsy was not performed.

## 3. Discussion

Sudden death from intracranial tumors is often associated with subarachnoid and/or cerebral hemorrhage from the tumor or intratumoral hemorrhage. Wakai et al. reported that hemorrhage from brain tumors was confirmed either clinically, surgically, or at autopsy in 45 of 1550 cases of brain tumors (2.9%) other than pituitary adenoma [2]. Hemangiopericytoma was not included in that series. According to the literature, among cases of sudden unexpected death arising from primary brain tumor, colloid cysts of the third ventricle are the most frequent, followed by gliomas, ependymomas, and meningiomas [6–10]. However, only three cases of sudden unexpected death arising from intracranial hemangiopericytoma have been reported, including the present case (Table 1) [4, 6, 11].

Brain tumor hemorrhage is caused by endothelial proliferation, which leads to vascular obliteration, vessel compression, and/or distortion as a result of rapid tumor growth resulting in distal necrosis and an increased venous pressure associated with increased intracranial pressure. In hemangiopericytoma, dilated vessels with thin walls including erosion, distortion, and/or distension of the blood vessels caused by tumor growth, in addition to intense vascularity of the tumor and changes in the structure of the vessel walls, are considered to be possible sources of hemorrhage. Bleeding might occur from the dilated tumor vessels near the dural attachment. In addition, coagulatory changes or fragile malformed tumor microvessels induced by radiotherapy may represent a cause of bleeding in some cases [12]. Weakening or malformation of tumor vessels may also be induced by radiotherapy, resulting in bleeding.

Mena et al. reported that 22 of 94 intracranial hemangiopericytoma cases (23.4%) exhibited intratumoral hemorrhage [13]. Cervoni et al. summarized and reviewed nine cases of meningeal hemangiopericytoma associated with subarachnoid [5], subdural [3, 14], intratumoral [12], and intracerebral hemorrhage [11, 15, 16]. All cases involved supratentorial lesions. According to the report from Maruya et al. [11], most patients with meningeal hemangiopericytoma associated with intracranial hemorrhage were men under 60 years old. Massive intracranial hemorrhage, especially from infratentorial lesions, resulted in rapid neurological deterioration and the need for emergent surgery. In the present case, the patient had already undergone craniotomy for total resection of an infratentorial hemangiopericytoma without adjuvant therapy such as radiation, differing from previously reported cases. Surprisingly, the tumor recurred at the same site with life-threatening massive intratumoral hemorrhage only 17 months postoperatively, representing a first report according to the literature. The cause of such rapid progression of recurrent hemangiopericytoma remains unclear. We speculated the pathogenesis as follows. Dilated tumor vessels at the dural attachment were only cauterized in the first operation, and residual tumor cells may have remained. Residual tumor cells at the dural attachment would then have rapidly progressed, and the tumor vessels were involved, resulting in fragile vessels.

Postoperative radiotherapy is generally recommended for hemangiopericytoma, particularly if the tumor is not removed completely. Several retrospective studies have demonstrated that radiotherapy after surgery is beneficial for patients with hemangiopericytomas [17–20]. In those studies, the mean local recurrence rate of patients with postoperative adjuvant radiotherapy was less than those without radiotherapy, suggesting that surgical removal followed by external radiotherapy reduced the risk of local recurrence. However, it was not demonstrated that postoperative radiotherapy protected against neuroaxis metastasis or peripheral metastasis [18]. Ghia et al. described that the extent of surgery and the use of adjuvant radiotherapy did not have statistically significant effects on overall survival [19, 20].

In the present case, we thought that gross total removal of the tumor had been successfully achieved; therefore, postoperative radiotherapy was not performed. However, the growth of the recurrent tumor in the present case might be generated from the enhancing lesion on the convexity dura at excision site, which grew more rapid than we had been afraid. Previous reports have described that mean time to local recurrence of hemangiopericytomas after initial surgery was between 40.5 to 104 months [20–23]. Jääskeläinen et al. described hemangiopericytomas attached to sinuses, occipitally located, and that bled profusely at operation had a higher risk of recurrence and metastasis [23].

In the present case, MIB-1 index in both initial and recurrent hemangiopericytomas was 3% and 5%, respectively, suggesting growth potential and suspicious recurrence of the tumor. In addition, regardless the extent of resection, patients with hemangiopericytomas have a high risk of developing tumor recurrence; thus, we should have considered postoperative radiotherapy for suspicious residual tumor on the convexity dura following the initial operation.

Sudden death from an intracranial tumor is often associated with a rapid increase in intracranial pressure as a result of hemorrhage from the tumor or acute obstructive hydrocephalus caused by obstruction of the flow of cerebrospinal fluid. In infratentorial tumors, direct involvement of the respiratory and cardiac centers is also postulated to be a further cause of sudden death [7]. Postoperative radiotherapy might be necessary, even if total removal of the tumor appears to have been achieved.

In conclusion, recurrent infratentorial hemangiopericytoma manifesting as intracranial hemorrhage is quite rare but carries a risk of life-threatening massive bleeding. Careful postoperative long-term follow-up by neuroimaging is very important to avoid delaying the diagnosis of local recurrence of hemangiopericytoma, particularly in the posterior cranial fossa.

## Figures and Tables

**Table 1:** 

Age/sex	Location	Treatment	Type of hemorrhage	Follow-up period between onset and death	Reference
9/M	Right lateral ventricle	Conservative	Intraventricular	4 days	Bunai et al. (2008) [4]
59/M	Right frontotemporal	Conservative	Intracerebral	9 days	Maruya et al. (2006) [11]
43/M	Right cerebellar hemisphere	Total removal	Intracerebellar	14 days	Present case
